# Stress and associated factors in public school teachers: a cross-sectional study

**DOI:** 10.47626/1679-4435-2022-832

**Published:** 2023-08-08

**Authors:** Lívia de Araújo Rocha, Andressa Santos de-Carvalho, Patrícia Regina Evangelista de-Lima, Bruna Araújo Gomes, Letícia Gonçalves Paulo, Rauene Raimunda de-Sousa, Luísa Helena de Oliveira Lima, Ana Roberta Vilarouca da-Silva

**Affiliations:** 1 Programa de Pós-graduação em Ciências e Saúde, Universidade Federal do Piauí (UFPI), Teresina, PI, Brazil; 2 Enfermagem, UFPI, Picos, PI, Brazil; 3 Departamento de Ciências e Saúde, UFPI, Teresina, PI, Brazil

**Keywords:** occupational stress, psychological distress, school teachers, estresse ocupacional, estresse psicológico, professores escolares

## Abstract

**Introduction:**

The search for improving productivity and performance at work has exposed workers to high levels of stress. Since the working conditions of basic education teachers interfere negatively with their health, it is important to study the entire context involving teachers, in order to encourage the promotion of workers’ health actions.

**Objectives:**

To investigate stress levels and associated factors in public school teachers.

**Methods:**

The study was conducted through online questionnaires sent to public school teachers in the state of Piauí, in 2020, using the snowball method. Sociodemographic and economic characteristics and risk habits (smoking, alcoholism, and sedentary lifestyle) of the study sample were investigated, as well as clinical, anthropometric, and stress-related conditions.

**Results:**

A total of 126 teachers participated in the study, most of which were women (88.9%), had a family income from three to four minimum salaries (30.2%), and worked two shift or more (55.6%); furthermore, 10.3% reported having hypertension; 8.7%, musculoskeletal diseases; 3.2%, thyroid-related problems; and 2.4%, diabetes. A statistically significant difference in median stress levels was observed in women (p = 0.002) and in individuals with thyroid problems (p = 0.015).

**Conclusions:**

Teachers, especially women working in this job, suffer from expressive levels of stress, which can directly affect their quality of life, requiring the development of measures to prevent stress, in order to improve the health and the performance of these professionals.

## Abstract (pt)

**Introdução:**

A busca por mais produtividade e desempenho no trabalho tem submetido trabalhadores a altos níveis de estresse. As condições de trabalho do professor de educação básica interferem de forma negativa sobre a sua saúde; assim, é importante que todo o contexto que envolve o professor seja estudado a fim de incentivar a promoção de ações de saúde do trabalhador.

**Objetivos:**

Verificar os níveis de estresse e fatores associados em professores de escolas públicas.

**Métodos:**

O estudo foi realizado através de questionários *on-line* enviados para professores de escolas públicas do Piauí, no ano de 2020, utilizando-se o método bola de neve. Foram investigadas as características sociodemográficas e econômicas, os hábitos de risco (tabagismo, alcoolismo e sedentarismo) e as condições clínicas, antropométricas e de estresse da amostra estudada.

**Resultados:**

Participaram deste estudo 126 professores, dos quais 88,9% eram mulheres, com renda familiar entre três e quatro salários-mínimos (30,2%), que trabalham em dois turnos ou mais (55,6%); 10,3% afirmaram possuir hipertensão, 8,7% disseram sofrer de doenças osteomusculares, 3,2% relataram problemas relacionados à tireoide e 2,4% são diabéticos. Foi encontrada uma diferença de medianas estatisticamente significante entre os níveis de estresse e o sexo feminino (p = 0,002) e problemas de tireoide (p = 0,015).

**Conclusões:**

Os professores, especialmente as mulheres, sofrem de níveis de estresse expressivos, o que pode afetar diretamente a qualidade de vida, sendo necessário que medidas preventivas de estresse sejam criadas a fim de melhorar a saúde e o rendimento desses profissionais.

## INTRODUCTION

Both stress and anxiety are understood as a response of our body to any threat to our integrity, in order to favor survival and adaptation to changes in our environment. However, this relationship may trigger several behaviors and diseases, impairing individual’s health.^1^

The continuous search for improving productivity and performance at work, for example, has exposed workers to high levels of stress and anxiety. Occupational stress can be caused by subjects’ perception of workplace actions and by their capacity to cope with them. In other words, in order for stress to occur, professionals have to evaluate the situation and the demands of the environment as stressors, and see themselves as having little capacity to deal with them, generating behaviors with a negative impact on their mental health.^2,3^

Some professional categories are more prone to experience stressful situations, such as teachers. The working process of an educator goes far beyond the classroom, and conditions involving teachers’ work, such as devaluation of their role within the society, low payment, as well as lack of recognition, may reflect directly on the mental health of these professionals, causing stress, vulnerability, and reduced professional skills.^4,6^

A study conducted with teachers^7^ observed that these professionals have significant rates of physical manifestations of stress, with one third of the sample showing psychological manifestations of stress above average stress scores. In another study that interviewed public school teachers, 72% of them answered that they considered their job stressful.^8^ Furthermore, public school teachers in the city of São Paulo, Brazil, participated in a study that found that 47.2% of the sample reported having a poor quality of life.^9^

Maintaining educators’ good health status is indispensable for their work to be properly performed.^5^ Thus, working conditions of basic education teachers are perceived to interfere negatively with their heath; hence, it is important to study the entire context involving teachers and their health so as to encourage the promotion of worker’s health actions, as well as improving understanding on stress-associated factors, in order to promote a discussion about the influence of work on the health of this population.

Therefore, the aim of this study was to investigate stress levels and associated factors in public school teachers in the state of Piauí, Brazil, to characterize this population according to sociodemographic, economic, behavioral, anthropometric, clinical, and stress-related variables, and identify the possible relationships between the observed characteristics and stress levels experienced by the sample.

## METHODS

### TYPE OF STUDY

This is an epidemiological, analytical, cross-sectional study with a quantitative approach. The study was conducted remotely, through online questionnaires sent by a smartphone messaging application to teachers from public schools located in the state of Piauí, Brazil.

### SAMPLE AND PATIENT SELECTION

The sample was obtained using the non-probabilistic exponential sampling technique known as “snowball,” which uses reference chains. Therefore, this method does not make it possible to determine the chance of participants to be selected for the research, but it is useful to study groups that are not easily accessible, such as teachers in pandemic times.^10^ In this method, an individual is invited and then indicates other people who can participate in the sample. The technique was applied until indications started to repeat themselves and when no more replies were obtained.^11^

The study included teachers of municipal or state public schools aged from 20 to 59 years who accepted to participate in the study. Workers who had some physical limitation that could modify self-reported anthropometric variables were excluded, as well as pregnant women or who had given birth less than 1 year before. A total of 126 teachers met inclusion and exclusion criteria.

### DATA COLLECTION AND STUDY VARIABLES

For data collection, teachers answered a questionnaire about their sociodemographic, economic, clinical, and anthropometric characteristics; moreover, stress levels were measured by the Perceived Stress Scale (PSS-14), and consumption of alcohol and physical activity levels were evaluated using the questionnaires The Alcohol Use Disorder Identification Test (AUDIT) and International Physical Activity Questionnaire version 8 (IPAQ-8), respectively.

### ETHICAL ASPECTS

The study was conducted after consideration by the Research Ethics Committee of Universidade Federal do Piauí (CEP/UFPI), via Plataforma Brasil, under no. 3.707.399. Furthermore, it complied with the Guidelines and Standards for Research Involving Human Beings, using Resolution no. 466/2012 of the National Health Council, which highlight the ethical aspects of research with humans. The participants who agreed to participate in the research received information on its objectives and justification and were invited to sign the Informed Consent Form (ICF).

### DATA ANALYSIS

For data analysis and curation, Pandas (version 1.1.5) and SciPy (version 1.4.1) libraries were used to formulate data structure and analysis for Python. A descriptive analysis was conducted by calculating absolute and relative frequencies of the studied variables, in addition to an analysis of means with 95% confidence intervals, as well as median with p25th-p75th and standard deviation.

The D’Agostino-Pearson test was applied to evaluate normality of distribution of quantitative variables. To verify significant differences in medians between sociodemographic and economic variables, risk factors, body mass index (BMI) and clinical conditions according to stress levels, non-parametric Mann-Whitney tests were applied for continuous variables and Kruskal-Wallis. For the correlation tests, the Spearman correlation coefficient was used to measure the strength of association between two variables. All analyses were performed considering a p-value below 0.05 (p ≤ 0.05) as statistically significant.

## RESULTS

Table 1 shows the characterization of the study sample, which predominantly consisted of women (88.9%), individuals self-reported as brown (56.3%), with a median age of 38.0 years, married (51.6%), and with complete higher education (67.5%). Most professionals who completed the questionnaires answered that they had been working as a teacher for more than 15 years (42.1%), worked in two shifts or more per day (55.6%), worked mainly in the first years of elementary education, and had a family income from 3 to 4 minimum salaries (30.2%).

**Table 1:** Sociodemographic and economic characterization of teachers, Picos, state of Piauí, Brazil, 2020 (n = 126)

Variables	n	%	Median (p25th-p75th)
Sex			
Female	112	88.9	
Male	14	11.1	
Skin color/ethnicity			
White	42	33.3	
Black	10	7.9	
Brown	71	56.3	
Others	3	2.4	
Age range (years)			38.0 (32.0-45.0)
18-23	6	4.8	
24-30	14	11.1	
31-35	26	20.6	
36-40	26	20.6	
> 41	54	42.9	
Marital status			
Single	40	31.7	
Married	65	51.6	
Divorced/separated	6	4.8	
Widower	2	1.6	
Stable union	13	10.3	
Family income (minimum salaries)			
1-2	25	19.8	
3-4	38	30.2	
5-6	32	25.4	
> 6	31	24.6	
Educational level			
Complete secondary school	6	4.7	
Complete higher education	85	67.5	
Incomplete higher education	-	-	
Non-graduate degree studies	31	24.6	
Graduate degree	4	3.2	
Work shift			
Morning	27	21.4	
Afternoon	24	19.0	
Evening	5	4.0	
Two shifts or more	70	55.6	
Time in the job (years)			
1-2	6	4.8	
3-5	15	11.9	
6-10	23	18.2	
11-15	28	22.2	
More than 15	53	42 .1	
Less than 1	1	0.8	

p25th-p75th = interquartile range.

With regard to the risk behaviors adopted by the sample, 98.4% of participating teachers reported not smoking, and 1.6% reported smoking 10 or more cigarettes a day. More than a half (54.6%) said that they did not consume alcoholic beverages, and 27.7% reported consuming once or twice a month. Most of the studied population had a low risk drinking behavior (95.2%). With regard to physical activity, 33.3% of the sample was classified as insufficiently active, whereas 20.6% were classified as sedentary, as shown in Table 2.

**Table 2:** Characterization of variables related to risk behaviors adopted by the sample, Picos, state of Piauí, Brazil, 2020 (n = 126)

Variables	n	%
Smoking		
Smoker	2	1.6
Non-smoker	124	98.4
Drinking behavior		
Low risk drinking	120	95.2
Risky or hazardous drinking	6	4.8
Harmful drinking	-	-
Likely alcohol dependence	-	-
Physical activity level		
Sedentary	26	20.6
Insuficiently active	42	33.3
Active	39	31.0
Very active	19	15.1

Table 3 reveals that most participants in this study had a normal range BMI (50.8%), whereas 27.8% were overweight and 3.2% had obesity grade 2. Of total participants, 10.3% reported having arterial hypertension; 8.7%, musculoskeletal diseases; 3.2%, thyroid-related problems; and 2.4%, diabetes. Mean height and weight of the sample were 1.60 cm and 65.3 kg, respectively.

**Table 3:** Clinical and anthropometric characterization of the sample, Picos, state of Piauí, Brazil, 2020 (n = 126)

Variables	n	%	Mean ± SD	95%CI
BMI (kg/m²)			25.19 ± 4.36	24.42-25.95
Underweight	4	3.2		
Normal range	64	50.8		
Overweight	35	27.8		
Obesity grade I	19	15.0		
Obesity grade II	4	3.2		
Obesity grade III	-	-		
Chronic diseases				
Yes	26	20.6		
No	100	79.4		
Hypertension	13	10.3		
Diabetes	3	2.4		
Musculoskeletal diseases	11	8.7		
Thyroid-related problems	4	3.2		

BMI = body mass index.

Table 4 shows the mean and median stress levels obtained with the PSS-14 answered by the study sample. Mean stress level was 26.32 ± 8.8, with a median of 26.0 points. In the studied scale, the higher the score, the stress level experienced by the individual.

**Table 4:** Stress levels according to Perceived Stress Scale (PSS-14), Picos, state of Piauí, Brazil, 2020 (n = 126)

Total score (mean ± SD)	Median
26.32 ± 8.88	26.0

Tests were conducted to compare median perceived stress levels with sociodemograhic, economic, clinical, and anthropometric data and risk habits adopted by the sample and, as shown in Table 5, it was found that women had higher median stress levels, as well as teacher who self-reported as black, married, who reported a family income above six minimum salaries, with complete secondary school, who worked in the morning shift, and those who had been working as a teacher from 3 to 5 years.

**Table 5:** Comparison between statistically significant median stress levels between studied variables, Picos, state of Piauí, Brazil, 2020 (n = 126)

Variables	Stress		
	Mean ± SD	Median (p25th-p75th)	p-value*
Sex			0.003
Female	27.1 ± 8.7	27.0 (22.0-30.0)	
Male	20.1 ± 7.5	18.5 (15.2-25.2)	
Skin color/ethnicity			0.666
White	27.1 ± 9.5	26.5 (21.0-30.0)	
Black	27.8 ± 5.1	27.5 (26.2-28.7)	
Brown	25.6 ± 9.0	26.0 (19.0-29.5)	
Others	26.6 ± 3.7	25.0 (24.0-28.0)	
Age range (years)			0.977
18-23	27.3 ± 8.2	26.0 (20.2-34.7)	
24-30	26.0 ± 7.4	27.0 (22.2-28.7)	
31-35	26.5 ± 6.2	27.5 (22.2-29.0)	
36-40	27.2 ± 11.6	25.0 (18.2-35.2)	
> 40	25.7 ± 9.0	26.0 (20.2-30.0)	
Marital status			0.799
Single	25.7 ± 8.6	24.0 (20.0-29.2)	
Married	26.5 ± 9.2	27.0 (21.0-30.0)	
Divorced	24.1 ± 4.3	25.0 (20.2-27.5)	
Widower	27.0 ± 1.4	27.0 (26.5-27.5)	
Stable union	28.1 ± 10.1	26.0 (22.0-35.0)	
Family income (minimum salaries)			0.679
1-2	24.7 ± 7.8	24.0 (20.0-28.0)	
3-4	26.8 ± 8.6	26.0 (22.0-30.0)	
5-6	26.2 ± 9.1	26.5 (18.7-31.0)	
> 6	27.0 ± 8.6	28.0 (22.0-30.0)	
Educational level			0.454
Complete secondary school	31.1 ± 13.3	29.0 (24.5-35.0)	
Complete higher education	25.6 ± 8.6	26.0 (21.0-29.0)	
Non-degree graduate studies	26.5 ± 8.7	27.0 (17.5-30.5)	
Graduate degree	31.0 ± 7.5	28.0 (26.0-33.0)	
Work shift			0.154
Morning	29.2 ± 9.6	27.0 (23.0-36.0)	
Afternoon	23.0 ± 8.5	21.5 (18.0-28.2)	
Evening	24.6 ± 4.5	26.0 (24.0-28.0)	
Two shifts or more	26.4 ± 8.6	26.5 (22.0-30.0)	
Time in the job (years)			0.436
1-2	21.3 ± 3.3	20.5 (19.2-21.0)	
3-5	29.7 ± 9.9	28.0 (22.0-37.0)	
6-10	26.7 ± 6.9	27.0 (23.5-29.0)	
11-15	25.5 ± 7.6	26.0 (20.7-28.5)	
More than 15	26.2 ± 10.2	26.0 (19.0-30.0)	
Less than 1	24.0 ± 0.0	24.0 (24.0-24.0)	
Smoking			0.791
Smoker	24.3 ± 12.0	24.5 (20.2-28.7)	
Non-smoker	26.3 ± 8.8	26.3 ± 8.8	
Drinking behavior			0.222
Low risk drinking	26.5 ± 8.9	26.5 ± 8.9	
Risky or hazardous drinking	21.5 ± 7.0	21.5 ± 7.0	
Harmful drinking	-	-	
Likely alcohol dependence	-	-	
Physical activity level			0.068
Sedentary	30.1 ± 10.6	29.0 (24.2-37.0)	
Insuficiently active	24.7 ± 8.7	24.0 (20.0-28.0)	
Active	25.8 ± 7.8	26.0 (21.0-29.5)	
Very active	25.4 ± 7.6	25.0 (20.0-28.5)	
BMI			0.891
Low weight	27.2 ± 7.7	25.5 (21.0-31.7)	
Normal weight	26.7 ± 9.3	26.0 (22.0-30.0)	
Overweight	26.7 ±9.5	28.0 (18.0-30.5)	
Obesity grade I	24.3 ± 6.9	26.0 (19.0-28.0)	
Obesity grade II	24.0 ± 4.8	25.0 (21.0-28.0)	
Obesity grade III	-	-	
Chronic diseases			0.384
Yes	27.9 ± 8.6	27.0 (22.5-30.7)	
No	25.9 ± 8.9	26.0 (20.7-29.2)	
Hypertension			0.649
Yes	26.6 ± 6.4	27.0 (22.5-30.7)	
No	26.2 ± 9.1	26.0 (21.0-30.0)	
Diabetes			0.229
Yes	36.0 ± 15.6	28.0 (27.0-41.0)	
No	26.0 ± 8.6	26.0 (20.5-30.0)	
Musculoskeletal diseases			0.975
Yes	25.8 ± 6.0	27.0 (22.5-29.0)	
No	26.3 ± 9.1	26.0 (21.0-30.0)	
Thyroid problems			0.015
Yes	37.0 ± 6.3	39.0 (34.7-41.2)	
No	25.9 ± 8.7	26.0 (20.2-29.0)	

* p ≤ 0.05.

BMI = body mass index.

Furthermore, higher median stress levels were observed in teachers with thyroid-related problems, diabetes, and overweight.

## DISCUSSION

Most participants in this study were women (88.9%), which is consistent with data from the Instituto Nacional de Estudos e Pesquisas Educacionais Anísio Teixeira (INEP) showing that 90% of teachers working in early childhood education are women; this percentage decreases in the final years of elementary education and in secondary school, but females are still predominant, accounting for 69 and 60% of teachers, respectively. The prevalence of women is typical of the studied category.^7,9,12,13,14^

In the present study, most teachers self-reported as brown (56.3%), 33.3% as white, and 7.9% as black, in line with other studies on the same subject, in which most teachers (47.3%) also self-reported as brown.^15^ Other studies showed different results, observing that most of the studied population self-reported as white, followed by black or brown.^13,14^

The teachers evaluated were predominantly aged above 41 years (42.9%). In the studies involving this category, it is possible to observe that there was a higher prevalence of professionals of more advanced age, coherent with the finding related to time working in schools, in which 42.1% of teachers reported working as a teacher for more than 15 years, and 22.2% for 11 and 15 years.

The majority of teachers participating in the present research reported working two shifts or more a day (55.6%). In a study conducted in Popayan, Colombia, 75% of teachers reported spending more than ¾ of their working hours interacting with students, whereas 43% reported working from 48 to 63 hours a week.^16^

The teachers studied here are mostly married (51.6%), with a family income from three to four minimum salaries (30.2%). With regard to teachers’ education, 67.5% stated to have complete higher education, 4.7% to have only complete secondary school, and 24.6% reported having attended non-degree graduate studies.

Of the teachers interviewed, 98.4% reported not smoking. Other studied found a prevalence of smoking of 4.4% in teachers.^14^ Moreover, 95.2% had a low-risk drinking behavior, and 20.6% were classified as sedentary. Overweight was present in 27.8% of the teachers, 15% had obesity grade I, and 3.2% had obesity grade II.

Nutritional status is strongly influenced by the extrinsic environment. Stress can cause an unbalance in the body, affecting individual’s food consumption. Stressful situations alter hormone levels, leading to chemical changes, such as the release of glucocorticoids (corticosteroids and adrenalin), which may cause eating disorders like obesity and stimulate the reward system, thus increasing binge-eating and therefore the intake of high-calorie foods, favoring weight gain.^17,18^

Additionally, work routine, extenuating working hours, lack of social support, and stress resulting from work activities are some factors that may related to teachers’ illness, influencing life habits and inappropriate food choices, which may lead to body weight gain and thus to the development of associated comorbidities.^13,19,20,21^

In a study that assessed the stress experienced by teachers,^18^ it was found that 67.9% of the study sample was overweight/obese, who showed higher stress levels compared with normal weight teachers, although this difference was not statistically significant. Similarly to the present study, median stress levels were slightly higher in overweight teachers.

The present study observed that the most common clinical conditions in the teachers studied were hypertension (10.3%), musculoskeletal diseases (8.7%), thyroid problems (3.2%), and diabetes (2.4%).

Another study also found that 81.5% of teachers reported having more than two diseases, and 31.5% of the sample classified their health status was poor or regular. Despite significant percentages, the diseases analyzed in the study were not related to stress.^18^

In a similar study, 20.67% of the teachers studied had hypertension, 19.82% had musculoskeletal diseases, and 5.55% had diabetes. The prevalence of obesity was 29% and was higher among women.^9^ A high prevalence of overweight/obesity (47.2%) was also observed in public school teachers in the state of Bahia, Brazil, but it was more prevalent among men.^15^

As previously seen, higher median stress levels were observed in women (p = 0.003), which may be partly explained by the fact that contemporary women are exposed to higher stress burden and have to deal with a large number of social demands. Moreover, they are definitely inserted into an increasingly more competitive labor market, in addition to housework, which may generate many conflicts.^22^ Because women have innate sensitivity, they are more susceptible to the detrimental effects of stress and to the development of related diseases.^23^

Female teachers experience greater perceived pressure in the workplace compared to male teachers.^13^ Tere is a naturalization of the idea that a less qualified work is performed by women, strengthening the view that work is an extension of their household activities.^24^ The pressure experienced by modern women may interfere with their physical and mental health, thus increasing stress levels.

Furthermore, higher stress levels were observed in teachers with thyroid problems in the study sample (p = 0.015). This relationship can be explained due to the fact that stress is capable of leading to the development of several health problems in individuals.^25^ Hyperactivation of the hypothalamus-pituitary-adrenal axis, caused by a hormone response to stress, generates a Th2-type immune response, suppressing cell immunity and increasing humoral immunity, which may beter elucidate the reason why autoimmune diseases, such as hyperthyroidism, are preceded by high stress levels.^26,27^

The hypothalamic-pituitary-thyroid axis, the central regulatory system that controls the production of thyroid hormones, is transitorily activated during acute stressful situations, whereas prolonged stress is associated with decreased activity of this axis.^28,29,30^

However, the present study found that the profession under study may be a source of stress, which may be related to the onset of several diseases such as those addressed here.

## CONCLUSIONS

An excessive amount of work activities demanded to teachers may be the cause of illness and consequent work leave among these professionals; therefore, it is important to assist this population closely and to create stress coping strategies in order to prevent the complications resulting from high stress levels, thus contributing positively to teachers’ quality of life.

Finally, it is suggested to conduct additional studies on the theme, aiming to make a robust and consistent analysis on what variables precede stress.
